# Electronic Health Record Burnout in Surgery and Strategies to Improve Burnout: A Narrative Review

**DOI:** 10.7759/cureus.98469

**Published:** 2025-12-04

**Authors:** Allison Karwoski, Nicholas Hricz, Kevin Schlidt, Yvonne M Rasko

**Affiliations:** 1 Department of Surgery, Division of Plastic Surgery, University of Maryland School of Medicine, Baltimore, USA; 2 Surgery, Sinai Hospital of Baltimore, Baltimore, USA

**Keywords:** burnout, documentation burden, electronic health record, physician well-being, surgery, usability

## Abstract

Physician burnout is a prevalent occupational syndrome characterized by emotional exhaustion, depersonalization, and diminished professional efficacy. Use of electronic health records (EHRs; also termed EMRs) has been associated with provider burnout in multiple settings. This review characterizes the most frequently reported EHR‑related contributors to burnout among U.S. surgeons across the following four domains: documentation burden, time demands (including after‑hours work), electronic messaging/in‑basket load, and usability. We searched PubMed for English-language, U.S. articles published from January 2004 to April 2024. Two reviewers independently screened and domain‑coded with consensus resolution. We report domain frequencies as n (%) with 95% confidence intervals (CIs) (Wilson) and summarize study‑level magnitudes where available (e.g., hours/day in EHR, after‑hours share, message volume, odds ratios). Of the 207 screened records, 25 were included across general, vascular, orthopedics, otolaryngology, surgical oncology, and the surgical intensive care unit. Domain frequencies: documentation, 15/25 = 60.0% (95% CI = 40.7-76.6); time demands, 17/25 = 68.0% (95% CI = 48.4-82.8); electronic messaging, 8/25 = 32.0% (95% CI = 17.2-51.6); and usability, 4/25 = 16.0% (95% CI = 6.4-34.7). None of the included studies explicitly assessed medicolegal contributors. Examples of magnitudes included EHR hours/day and remote/after‑hours EHR use, message volumes and timing, and fatigue/efficiency markers. In U.S. surgical literature, EHR‑related burnout is most frequently reported in association with documentation burden and time demands, with additional contributions from electronic messaging and usability. Practical mitigation includes surgeon‑facing efficiency tactics (templates, dictation, team inbox protocols) alongside system‑level usability improvements and policy reforms. Further prospective and interventional evaluations are needed.

## Introduction and background

Electronic health record (EHR) systems, also termed electronic medical records (EMRs), were introduced to improve clinical efficiency, accessibility, and reliability of patient information [1]. First introduced in the 1970s, EHR use has increased steadily, with broad adoption across U.S. hospitals and office-based practices [2]. EHRs have been linked to increased adherence to evidence-based care and safety improvements, including fewer medication errors, fewer unnecessary tests, improved anticoagulation prophylaxis adherence, increased vaccination rates, and lower mortality [3-7]. Recent narrative reviews of physician and medical student burnout further underscore the need for multilevel strategies to address this syndrome [8]. However, integration into clinical workflow can be associated with burnout among surgical professionals. The magnitude of this association varies by specialty, role, and study design [9-11].

Physician burnout is a prevalent syndrome with components of depersonalization, exhaustion, and a diminished sense of accomplishment [11]. In repeated surveys of U.S. physicians, burnout prevalence increased from ~35% to ~56% in some samples between 2013 and 2020 [12-15]. Surgeons have elevated risk and consequences, including substance misuse, attrition, interpersonal strain, suicidal ideation, depression, and potential impacts on patient outcomes [16-19]. Consistent with observational evidence, we use association language throughout and pre-specify four domains to align the Introduction and Results: documentation burden, time demands (including after-hours use), electronic messaging/in basket load, and usability [9-11]. We also note that well‑implemented EHR features (e.g., standardized order sets, dashboards, team-based messaging rules, voice dictation) can reduce friction in some workflows. Because EHR configurations, documentation requirements, and policy incentives differ across countries, we focused on U.S.-based studies to provide a coherent and policy-relevant synthesis. Our objective was to characterize the most frequently reported EHR-related contributors to burnout among U.S. surgeons across these four domains and outline practical, evidence-aligned strategies.

## Review

Methodology

Search Criteria

We searched PubMed on April 30, 2024, for studies from January 1, 2004, through April 30, 2024. A Preferred Reporting Items for Systematic reviews and Meta-Analyses literature search extension (PRISMA‑S) compliant strategy with Boolean terms, MeSH, date limits, and filters appears in Appendix 1. In brief, we combined EHR/EMR terms (“Electronic Health Records”[Mesh] OR EHR* OR EMR* OR “electronic medical record*” OR “health information technolog*”), burnout/stress terms (“Burnout, Professional”[Mesh] OR burnout OR stress OR workload OR “time demand*” OR inbox OR messag* OR usabilit*), and surgical terms (“Surgery”[Mesh] OR surg* OR otolaryngology OR orthopedic* OR vascular surgery OR surgical oncology OR intensive care OR ICU). Limits: English; Humans; 2004-2024. We hand‑searched reference lists of included articles. No protocol was registered (e.g., Open Science Framework); this is stated as a limitation.

Inclusion and Exclusion Criteria

**Figure 1:**
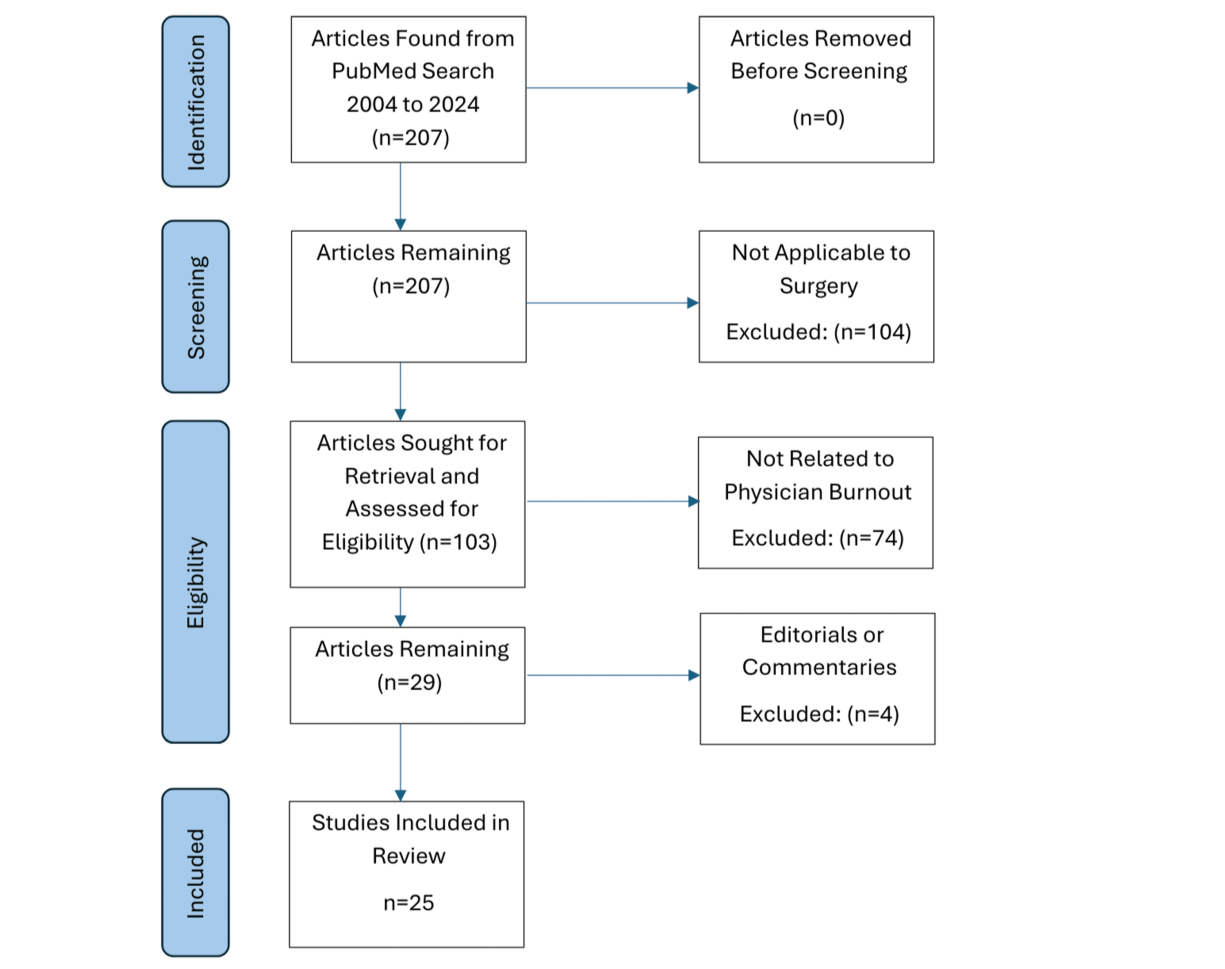
Reporting Items for Systematic reviews and Meta-Analyses flowchart of study selection.

We included U.S.‑based English-language studies published from 2004 to 2024, addressing surgeons/surgical trainees/surgical services and reporting EHR‑related burnout or stress/workload linked to burnout (qualitative or quantitative). We excluded non-surgical, non-burnout-related editorials/commentaries. Mixed populations (e.g., intensive care unit (ICU) including non‑surgeons) were included if informative to surgical workflows and flagged in text. A total of 207 records were screened, of which 25 met the inclusion criteria (Figure 1).

Data Extraction, Coding, and Inter‑rater Reliability

Eligibility was assessed by the first and second authors. Two authors independently coded whether each of the four domains (documentation, time demands, electronic messaging, usability) was reported as a contributor to burnout. A codebook (Appendix 2) defined each domain and decision rules for “contributor” (1), “assessed but not contributor” (0), and “not assessed” (NA) with verbatim criteria/examples. We computed percent agreement (P₀) and Cohen’s kappa (κ) for each domain, excluding NA codes. Agreement was moderate across domains (κ = 0.38-0.61).

Summary Methods and Statistics

Given heterogeneity, we conducted a structured narrative synthesis. To provide statistical context beyond counts, we report domain frequencies as n (%) with 95% Wilson score confidence intervals (CIs). Where available, we also summarized key design features, burnout measures, and EHR exposure definitions for each included study in Appendix 3 as a brief methodological appraisal; we did not apply a formal risk-of-bias scoring tool. Sensitivity tallies limited to validated burnout measures (e.g., Maslach Burnout Inventory (MBI)) and comparable exposure metrics (e.g., after‑hours %) were pre‑specified but constrained by reporting heterogeneity; we describe these plans in Appendix 2 and temper conclusions accordingly.

Results

We screened 207 records, of which 25 studies met the inclusion criteria for this narrative synthesis (Figure 1). Studies spanned general surgery, vascular surgery, orthopedics, otolaryngology, surgical oncology, and surgical intensive care (SICU). Sample compositions included attending surgeons, surgical residents, and mixed surgical services.

Coding Approach and Table Conventions

Each included article was domain‑coded for whether it reported an EHR‑related contributor to burnout in four a priori domains, namely, documentation burden, time demands (including after‑hours/remote use), electronic messaging/in‑basket load, and usability. In Table 1, an “X” denotes that the domain was reported as a contributor in that study; empty cells indicate the domain was assessed but not identified as a contributor. A brief verbatim‑trigger codebook (criteria/examples) is shown in Appendix 2.

**Table 1:** Included studies and electronic health record-related domains reported as contributors to burnout (n = 25). X = domain reported as contributor to burnout in that study; empty cell = domain assessed but not identified as a contributor; NA = not assessed; NR = assessed as a contributor but not reported quantitatively (not observed in these 25 studies).

Source (authors, year; ref)	Potential source of burnout			
	Documentation burden	Usability	Time demands (including after-hours/remote use)	Electronic messaging
Malay, 2020 [20]	X	NA	X	NA
Cox et al., 2021 [21]	NA	X	X	NA
Ebbers et al., 2022 [22]	X	X	X	NA
Dymek et al., 2021 [23]	X	NA	NA	X
Yan et al., 2021 [24]	X	NA	X	X
Lilly et al., 2019 [25]	NA	NA	X	NA
Kesler et al., 2022 [26]	NA	NA	X	X
Somerson et al., 2020 [27]	X	NA	X	NA
Victores et al., 2014 [28]	NA	NA	X	NA
Coleman et al., 2021 [29]	X	NA	X	NA
McPeek-Hinz et al., 2021 [30]	X	NA	X	NA
Lin et al., 2021 [31]	NA	X	NA	NA
Bahr et al., 2023 [32]	NA	X	NA	NA
Ho et al., 2023 [33]	X	NA	X	NA
Drudi et al., 2022 [34]	X	NA	NA	NA
Carayon et al., 2015 [35]	X	NA	X	NA
Aziz et al., 2019 [36]	X	NA	X	X
Wormer et al., 2015 [37]	X	NA	X	NA
Ham et al., 2016 [38]	X	NA	X	NA
Shenson et al., 2016 [39]	NA	NA	NA	X
Sun et al., 2018 [40]	NA	NA	NA	X
Cronin et al., 2015 [41]	NA	NA	NA	X
Congelosi et al., 2023 [42]	NA	NA	X	X
Crowson et al., 2016 [43]	X	NA	X	NA
Freiburg et al., 2011 [44]	X	NA	NA	NA

Domain Frequencies With Confidence Intervals

Across the 25 studies, the most frequently reported EHR‑related contributors were time demands and documentation, followed by electronic messaging and usability (Table 2). We present Wilson 95% CIs to add statistical context to simple tallies.

**Table 2:** Frequency of electronic health record-related domains reported as contributors to burnout across included studies (n = 25). CI = confidence interval

Domain	Studies reporting as contributor (n/N)	% of studies	95% CI (Wilson)
Documentation burden	15/25	60.0%	40.7–76.6%
Time demands	17/25	68.0%	48.4–82.8%
Electronic messaging	8/25	32.0%	17.2–51.6%
Usability	4/25	16.0%	6.4–34.7%

Stratified Descriptive Summaries

Given heterogeneous designs and reporting, we provide descriptive stratifications (see Appendix 3 for study-level details by specialty, role, setting, and era).

By specialty (general/vascular/orthopedics/ENT/oncology/SICU): In each specialty cluster, time demands and documentation remained the top two reported domains. Messaging burden appeared most often in services with high outpatient follow‑up and portal use; usability issues surfaced around transitions and downtime.

By role (attending vs. resident): Residents more often reported after‑hours EHR time and duty‑hour implications; attendings more often reported remote/nighttime EHR use and in‑basket load.

By setting (outpatient vs. inpatient/ICU): Outpatient settings emphasized documentation and messaging, whereas ICU/inpatient settings emphasized task volume, task switching, and workflow complexity within the EHR.

By era (2004-2012, 2013-2019, 2020-2024): Studies from 2020-2024 more frequently mentioned portal messaging growth and remote access patterns; the overall rank order of domains (time, documentation > messaging > usability) did not change directionally.

Sensitivity Notes

Where studies used validated burnout instruments (e.g., MBI) or comparable exposure metrics (e.g., hours/day in EHR, after‑hours %), the directional pattern (time/documentation most frequently reported) was unchanged. Because of small strata and reporting heterogeneity, we did not perform inferential comparisons or meta‑analysis of effect sizes. Selected study‑level magnitudes (e.g., EHR hours/day, remote use share, odds ratios linking inbox volume or documentation time to burnout) are summarized in Appendix 3 for context.

Other Observations

None of the included studies explicitly assessed medicolegal contributors to burnout; this likely reflects the scope of the search and study aims rather than proof of no effect.

Discussion

We identified 25 U.S. studies relevant to EHR‑related burnout in surgical settings spanning general surgery, vascular surgery, orthopedics, otolaryngology, surgical oncology, and ICU care. Across these studies, the most frequently reported contributors were time demands and documentation burden, with additional contributions from electronic messaging and usability (Tables 1, 2). To avoid over‑interpretation of heterogeneous observational evidence, our statements reflect associations rather than causation. Where available, we summarize study‑level magnitudes (e.g., hours/day in the EHR, after‑hours share, message volume, odds ratios) without aggregating across unlike populations or settings. None of the included studies explicitly assessed medicolegal contributors; this likely reflects scope/search limits rather than the absence of an effect. Finally, we direct readers to Table 2 (domain frequencies with 95% CIs) and Appendix 3 (select effect estimates) for a statistical context that goes beyond simple tallies.

Documentation

In U.S. surgical practice, documentation burden is consistently associated with burnout signals. Studies describe long, billing‑oriented notes, copy‑forward content, and extensive data entry that enlarge cognitive load and divert attention from patient‑facing work [45-47]. For context, ambulatory time‑motion data show substantial portions of the workday devoted to EHR/desk tasks; specialty and oncology clinics similarly report intensive documentation effort during visits. We emphasize that these estimates come from different roles and settings (e.g., ambulatory attendings vs. disease‑specific clinics) and therefore are not additive. Together, they support the qualitative pattern that documentation processes, i.e., note composition, order entry, and chart review, are frequently cited alongside burnout measures in surgical environments [48-54].

Time Demands

Surgeons have the highest mean annual hours worked of all specialties (+303 hours over average); additional time spent navigating EHRs detracts from other patient care, self-care, or other important life events [55]. One study found that surgeons, when they fail to balance administrative and clinical demands with their personal lives, gave their personal lives a lower priority [56]. Across several included studies, approximately 17% of EMR encounters occurred outside of scheduled working hours, underscoring the extent of after-hours work [22,32,57]. EMR usage at home was associated with increased odds of burnout [53]. In a time‑use analysis of academic surgeons, roughly 35% of EMR time occurred remotely, with a greater proportion of this time occurring during nighttime hours and on Sundays [21]. Because these estimates come from distinct systems, roles, and measurement approaches, they should not be interpreted as additive; rather, they converge on the pattern that EHR‑related tasks frequently extend into personal time.

Electronic Messaging

Every surgical specialty has experienced growth in outpatient interactions, commonly through electronic messaging [39,41]. While there are positive benefits to electronic messaging, such as the ability to mitigate patient frustrations, concerns, and questions while decreasing costs, over-utilization can also impact burnout. High patient call message volumes are associated with higher rates of provider burnout [24]. Electronic messaging between healthcare providers has also increased. While messaging allows for effective relaying of information, inappropriate messaging unnecessarily burdens providers. In one tertiary academic hospital, roughly 28% of nighttime communication was classified as non-urgent, highlighting the importance of timing and content [40].

Usability

Transitioning to use EHRs or transitioning from a different EHR system can cause struggles for providers [2]. While certain basic principles exist between them, there can also be variability within the same EHR based on the hospital. Despite this, little literature exists to guide EHR transitions, instead relying on the internal training teams when applicable [58]. Additionally, for providers who are required to learn how to operate EHRs for the first time, their efficiency drastically drops. While it does eventually improve, it often does not return to the pre-EHR baseline [37]. One limitation of EHRs also relates to downtime, the period when the system is partially or fully unavailable. These time periods can pose significant risks to patients and hospital operations. While protocols often exist to mitigate this risk, one study showed that the protocols were only executed 27.6% of the time [59]. Providers who are dissatisfied with their EHR are at a higher risk of burnout; however, satisfaction with one’s EHR was found to be protective of burnout, demonstrating the difference that a functional, usable EHR makes [34]. In a large nurse survey linked to hospital outcomes, poor EHR usability was associated with higher intention to leave among nurses and with significantly higher odds of inpatient mortality and 30-day readmission [60].

Burnout Strategies

Surgeons’ workloads are already high between balancing scheduled surgery, clinic patients, consultations, administrative tasks, hospitalized patient visits, and emergent surgeries. Given the already high risk of burnout in the field, combating the issue from every avenue is important [15]. While global changes to EHRs would likely have the most significant contribution to EHR-related burnout rates in surgeons, the changes will take time and likely require years of refinement to ultimately achieve the desired outcome. The design of EMRs would ideally be optimized on a user-centered design with direct input from physicians. By creating a system that enhances usability and patient care, physicians would be more likely to adopt and optimize an EHR [61]. While EHRs do have limitations, they also provide an economic advantage, increased quality of care, and can be a protective factor against burnout when utilized well [31,34,62].

The quality of EHR training can impact usability, which can then be extrapolated to improved burnout rates [63]. Most surgeons likely remember their onboarding training at a new hospital included hospital tours, meeting staff, company policies, going through multiple handbooks, and, at some point, going through the EHR. Surgeons frequently receive suboptimal introductions to their new practice settings; robust onboarding can help mitigate the transition [64]. This extends beyond index training, as one study evaluated 1,010 providers participating in a personalized re-training program designed to improve efficiency and knowledge using EHRs. The results showed a reduction in burnout rates from 32% to 23% [65]. Studies have also shown that daily EHR usage decreases over a period of years, highlighting that physicians also improve their efficiency over time [33].

Speech recognition software allows physicians to create text from voice commands, decreasing documentation time [66]. This technology has continued to become more complex and useful in documentation [23,67]. Artificial intelligence (AI) has the potential to drastically impact documentation by leveraging natural language processing technology to automate documentation of patient visits in EHRs [68]. The benefits of AI, particularly regarding machine learning, are not limited to documentation only. While still in its infancy, rapid processing and analysis of past medical experiences can be an excellent therapeutic aid. Although it is unlikely to fully replace a surgeon’s own onus to perform a chart review, it may develop into an adjunct that decreases EHR-related time.

Medical scribes have been proposed to alleviate stress through decreased provider-EHR time, increased patient and provider satisfaction, improved workflow efficiency, and billing and reimbursement coding optimization [47,69,70]. Systematic reviews and meta-analyses support scribe usage with an improved impact on relative value unit per patient hour as well as satisfaction [71]. While the impact on burnout has some varied data, some studies show reduced burnout and significantly less time charting [72]. One retrospective study examined 148,410 scribed encounters at 55 different clinics and found that surgery had the lowest median completion time, further showing the potential for scribes to improve documentation-related burnout [47]. These findings had a financial impact, as one pediatric plastic surgery clinic at a tertiary care facility found that scribes reduced their cost per office visit [73]. With an improvement in documentation burden, coding, and inter-office communication, scribes may be a useful addition to help mitigate burnout [74]. At the individual level, surgeons can also adopt practical tactics to use the EHR more efficiently and reduce burden. Examples include customizing templates and order sets for common scenarios, using speech recognition or macros for repetitive documentation, batching inbox and portal message review into scheduled blocks, delegating appropriate tasks through team-based messaging protocols, and requesting optimization or retraining sessions when workflows change. These strategies cannot fully offset structural and policy constraints but provide pragmatic steps within current systems.

While some strategies above can help mitigate burnout related to EHRs in the surgical field, the largest and most lasting change would have to be at a policy level. While vendors can mitigate difficulties with current issues, until it is required that EMRs are produced in a user-friendly design, with input from healthcare professionals, have interoperability standards to easily facilitate the exchange of information between systems, and there is a reform to payment policy to include care coordination and accuracy of information provided, there is little incentive for EHRs to adapt to the needs of patients [46]. The requirement of significant changes to the usability of EHR, including reform of certification standards, interoperability, and increasing physician engagement in the design, implementation, and customization, would be an important first step in addressing EHR-related issues.

While EHRs have made a substantial positive contribution to healthcare, they have also been frequently associated with physician burnout in surgical specialties. This review of the literature identified documentation and time demands as the two most frequently reported EHR‑related domains associated with burnout signals. Surgeons are inherently placed under an immense amount of stress at baseline in their careers, and the inherent risk of burnout associated with providing complex surgical care needs to be better addressed. Ultimately, for there to be a substantive impact on burnout rates, changes to EMRs need to happen at a policy level. However, steps can be taken in the interim to attempt to better mitigate burnout. Addressing EHR-related burden is essential to sustaining the surgical workforce and ensuring safe, efficient patient care.

Limitations

This review is limited by a single database (PubMed) search, restriction to U.S.‑based, English‑language studies, and lack of a formal risk‑of‑bias assessment, which may exclude relevant international or non‑English literature and limit comparability across heterogeneous designs and measures. As a narrative (rather than systematic) review, heterogeneity in study designs, burnout measures, and EHR exposure definitions precludes meta‑analysis and formal pooling. Selection and publication bias are possible. Specialty‑specific workflows and local EHR configurations may restrict generalizability across surgical settings. To enhance reproducibility, we provide PRISMA‑S search details and a brief domain codebook; discrepancies in domain coding were resolved by consensus.

## Conclusions

This narrative review of 25 studies highlights that EHR-related burnout among surgeons is most frequently reported in association with excessive documentation demands and time burdens, with additional contributions from electronic messaging and usability issues. Although EHRs have improved safety, coordination, and transparency in patient care, they have also introduced significant administrative strain that detracts from efficiency and work-life balance. Addressing this issue will likely require a multifaceted approach, enhancing system usability, optimizing workflow integration, expanding effective onboarding and retraining programs, and supporting the adoption of tools such as speech recognition, AI, and medical scribes. Lasting improvement will probably depend on policy-level reforms that promote user-centered EHR design and interoperability standards. Given the observational and heterogeneous nature of the available evidence, these findings should be interpreted as associations rather than causal effects; future comparative, prospective, and interventional studies will be important to test whether targeting these domains reduces burnout and improves outcomes. Mitigating EHR-related burnout is critical to preserving surgeon well-being, sustaining workforce longevity, and ensuring the delivery of safe, high-quality surgical care.

## Appendices

 Appendix 1

**Table 3:** PubMed search strategy (Preferred Reporting Items for Systematic reviews and Meta-Analyses literature search extension (PRISMA‑S)).

Item	Description
Database	PubMed
Date last searched	April 30, 2024
Time period	January 1, 2004 to April 30, 2024
Population/Context	Surgeons, surgical trainees, surgical services (general surgery, vascular surgery, orthopedics, otolaryngology, surgical oncology, surgical intensive care / ICU
EHR/EMR search terms	“Electronic Health Records”[Mesh] OR EHR* OR EMR* OR “electronic medical record*” OR “health information technolog*”
Burnout/Stress terms	“Burnout, Professional”[Mesh] OR burnout OR stress OR workload OR “time demand*” OR inbox OR messag* OR usabilit*
Surgical terms	“Surgery”[Mesh] OR surg* OR otolaryngology OR orthopedic* OR vascular surgery OR surgical oncology OR intensive care OR ICU
Boolean combination	“Electronic Health Records”[Mesh] OR EHR* OR EMR* OR “electronic medical record*” OR “health information technolog*”) AND (“Burnout, Professional”[Mesh] OR burnout OR stress OR workload OR “time demand*” OR inbox OR messag* OR usabilit*) AND (“Surgery”[Mesh] OR surg* OR otolaryngology OR orthopedic* OR vascular surgery OR surgical oncology OR intensive care OR ICU)
Limits/Filters applied	Humans; English language; publication date from 2004/01/01 through 2024/04/30
Additional steps	Hand-search of reference lists of included articles; exclusion of editorials/commentaries and non-surgical studies at screening/eligibility stages

 Appendix 2

**Table 4:** Codebook for electronic health record (EHR)-related burnout domains and coding rules.

Domain	Operational definition	Typical triggers	Coding values and rules
Documentation burden	EHR-related documentation tasks perceived as excessive, duplicative, or low-value, or explicitly linked to stress, workload, or burnout among surgeons or surgical teams. Includes note-writing, templating, copy-forward, billing-oriented documentation, and complex order entry	“Long, billing-oriented notes”; “excessive documentation time”; “increased clerical burden”; “copy-paste, copy-forward”; “charting burden”	1 = contributor: study qualitatively or quantitatively links documentation burden to burnout, stress, or workload. 0 = assessed but not contributor: documentation is measured or described but not associated with burnout signals. NA = not assessed: documentation burden not described as an exposure or theme. NR = assessed but not reported quantitatively: documentation burden discussed as contributor but without numeric reporting (e.g., only narrative comments)
Time demands (including after-hours)	EHR-related time load, including total hours/day in the EHR, share of time on EHR tasks during clinical sessions, and after-hours or remote use (evenings, nights, weekends). Includes duty hour implications and work-life boundary issues explicitly tied to EHR use	“Hours per day in the EMR”; “17% of encounters outside working hours”; “one fifth of EHR time after duty hours”; “EHR use at home”; “time pressure”	Coding as above (1, 0, NA, NR). 1: if the paper links EHR-related time demands (especially after-hours or remote use) to burnout, stress, or workload among surgeons/surgical teams 0: if measured but not linked NA: if not discussed NR: if only qualitatively described
Electronic messaging/Inbox load	Patient portal messages, secure messaging, inbox volume, call/message burden, and night-time communications (pages, alerts) handled via or tightly coupled to the EHR, where these are explicitly discussed as contributors to workload, stress, or burnout	“High patient call message volumes”; “secure messaging through patient portal”; “non-urgent pages at night”; “inbox burden”; “message volume”	Coding as above (1, 0, NA, NR). 1: if message/inbox burden is linked to burnout, stress, or workload 0: if messaging is measured but not associated with burnout NA: if messaging not discussed NR: if described as a burden but only narratively (no numeric volume or association)
Usability	Perceived EHR usability (navigation, layout, clicks, cognitive load), system transitions (switching EHRs), downtime, training quality, and user satisfaction, where these are explicitly tied to fatigue, stress, burnout, or intent to leave among clinicians caring for surgical patients	“Poor EHR usability”; “difficulty with EHR transition”; “downtime protocols not followed”; “EHR satisfaction protective”; “technostress”	Coding as above (1, 0, NA, NR). 1: if usability or satisfaction with the EHR is clearly linked to burnout, stress, fatigue, or turnover intent 0: if usability is measured but not associated with burnout NA: if usability not discussed NR: if only qualitatively linked without numeric reporting or effect estimate

 Appendix 3

**Table 5:** Methodological summary of included studies and selected electronic health record (EHR)-related magnitudes.

Source (authors, year; ref)	Specialty/Population	Primary role (s)	Design/Data source	Burnout/Stress Outcome	EHR-related exposure/metric	Key quantitative findings
Malay, 2020 [20]	Foot & ankle/podiatric surgery	Surgeons	Commentary/Perspective	Conceptual link to stress/burnout	Expansion and complexity of the medical record; growing documentation and chart length	No empirical data; conceptual argument that documentation burden and record growth contribute to workload and stress
Cox et al., 2021 [21]	Academic general surgery attendings	Surgeons/Attendings	Retrospective EHR login log analysis	Workload/Time only	EHR login time by day; clinic vs. operating room (OR) vs. administrative days; on-site vs. remote use	51 attendings logged mean 2.0 h/day and 13.8 h/week in the EHR; top 15% used EHR 4.6 h/weekday; 65% of EHR time on-site and 35% remote; remote use concentrated at night and on Sundays
Ebbers et al., 2022 [22]	Head & neck cancer care (oncology clinics)	Mixed providers, including surgeons	Cross-sectional time motion study	Documentation burden, perceived stress	Time in EHR per consultation; mouse events; keystrokes; perceived adequacy of documentation time	EHR tasks comprised 44.0% of initial consultation and 30.7% of follow-up consultation time; 593 mouse events and 1,664 keystrokes per initial consult; 140 mouse events and 597 keystrokes per follow-up; ≈13 clicks and 40 keystrokes per minute; <25% of providers felt there was enough time for documentation
Dymek et al., 2021 [23]	Mixed surgical specialties	Physicians, surgeons	Concept/Policy report	Time burden	Identifies domains of EHR-related clinician burden (documentation, inbox, usability, etc.)	No numeric effect estimates; synthesizes existing evidence and policy directions to reduce EHR-related burden
Yan et al., 2021 [24]	Mixed surgical specialties	Physicians, surgeons	Systematic review (26 studies)	Burnout with validated instruments	EHR-specific risk factors: insufficient documentation time; high inbox/patient call volume; negative EHR perceptions	Across included studies, insufficient time for documentation associated with burnout (OR = 1.40–5.83); high inbox or call volumes (OR = 2.06–6.17); negative EHR perceptions (OR = 2.17–2.44)
Lilly et al., 2019 [25]	Intensivists/ICU physicians	Intensivists	Observational + EHR task analysis	Burnout prevalence (self-reported)	Volume and length of documentation; ICU task load derived from EHR; mandatory task time vs. scheduled time	Reports a direct association between increases in documentation length at EHR implementation and increases in self-reported burnout; ICU workload judged excessive relative to scheduled time (no specific ORs in abstract)
Kesler et al., 2022 [26]	Orthopedic surgeons vs. mixed surgical specialties	Orthopedic surgeons, other surgeons	Retrospective EHR usage log	HER burden as a burnout proxy	Time on messages, note writing, chart review, orders, imaging review; % of scheduled clinic day spent on EHR	Orthopedic surgeons saw ≈31 pts/office day vs. 18 (other surgeons) and 12 (nonsurgeons); EHR tasks (messages, notes, review, orders) consumed 58% of an orthopedic surgeon’s clinic day; orthopedic surgeons received more EHR messages but answered them more efficiently than comparators
Somerson et al., 2020 [27]	U.S. orthopedic surgery residents	Residents	Cross-sectional online survey	Burnout (Maslach Burnout Inventory)	Work hours per week; EMR use >20 h/week; verbal abuse; support variables	Burnout prevalence 38%; working >80 h/week associated with burnout (OR = 2.8, 95% CI = 1.1–7.8); EMR use >20 h/week (OR = 2.1, 95% CI = 1.0–4.5); verbal abuse more than rarely (OR = 3.7, 95% CI = 1.3–11.5); adequate nursing support protective (OR = 0.2, 95% CI = 0.04–0.5)
Victores et al., 2014 [28]	Otolaryngology residents	Residents	Time motion study	Workload/Efficiency	Time spent in EHR vs. non-EHR tasks during inpatient service; order entry; note writing; chart review	Quantified resident time allocation among EHR tasks and other activities; detailed percentages and times not captured in abstract here (no burnout measure)
Coleman et al., 2021 [29]	Vascular surgeons	Practicing vascular surgeons	Cross-sectional wellness/burnout survey	Burnout and wellness metrics (validated survey items)	Multiple contributors, including workload, call, administrative tasks; EHR-related documentation and time demands considered among stressors	Reports burnout prevalence and predictors among vascular surgeons; administrative/EHR-related burdens identified qualitatively as contributors; specific EHR-related ORs not provided in abstract
McPeek-Hinz et al., 2021 [30]	Mixed specialties	Mixed providers, including surgeons	Cross-sectional survey	Burnout (self-report)	Work culture, clinician type, sex, and EHR use characteristics (e.g., satisfaction, perceived burden)	Demonstrates associations between EHR use and burnout alongside work culture and clinician factors; specific EHR-related effect sizes not reported in the abstract
Lin et al., 2021 [31]	Mixed hospital clinicians	Mixed providers, including surgeons	Observational EMR vs. non-EMR comparison	Healthcare quality	EMR implementation status vs. process and outcome quality indicators	EMR use associated with improved quality measures (e.g., processes, outcomes); study cited as context for potential quality benefits; no burnout metrics
Bahr et al., 2023 [32]	Physicians (various specialties)	Mixed providers, including surgeons	Cross-sectional online survey	Burnout (Maslach Burnout Inventory)	Work-related ICT use outside of working hours (not limited to EHR); years in practice	Work-related technology use outside working hours and years in practice were significant predictors of burnout in multivariable models; specific coefficients not given in the abstract
Ho et al., 2023 [33]	Vascular surgery residents	Residents	Observational EHR log analysis	Workload/Efficiency	Remote access to EMR vs on-site-only use; overall EMR time; distribution of EMR work	Remote EMR access was associated with reductions in overall EMR time for vascular surgery residents; exact time differences not reported in the abstract
Drudi et al., 2022 [34]	Practicing American vascular surgeons	Attending vascular surgeons	Cross-sectional gender-based survey	Burnout and related outcomes	Multiple potential predictors, including workload, work environment, and system factors (HER / administrative burden included among predictors)	Identified predictors and sequelae of burnout; EHR-related factors discussed among practice stressors; specific EHR-related effect estimates not provided in the abstract
Carayon et al., 2015 [35]	ICU residents and attendings	Residents and attendings	Pre/post time-motion observation	Workflow outcomes	Time spent in clinical review and documentation before vs. after EHR; task switching frequency; temporal flow of tasks	After EHR implementation, residents’ and attendings’ time on clinical review + documentation increased by 40% and 55%, respectively; resident task switches rose from 117 to 154 tasks/hour; attending switches fell from 138 to 106 tasks/hour
Aziz et al., 2019 [36]	Vascular surgery residents	Residents	Observational log time analysis	Workload proxy	EHR use during and after duty hours; share of EHR time outside scheduled duty	Residents spent approximately 20% of their EHR time after duty hours (per title); supports the characterization of substantial after-hours EHR work
Wormer et al., 2015 [37]	General surgery residents	Residents	Pre/post implementation observational study	Workflow, duty hours, operative experience	Introduction of EHR; resident workflow; duty hours; operative case volume	EHR implementation altered resident workflow, duty hours, and operative experience; detailed time and case metrics not reported in the abstract used here
Ham et al., 2016 [38]	General surgery residents	Residents	Pre/post intervention study	Efficiency and duty hours	EMR-based rounds report; resident efficiency; time for direct patient care and education; duty-hour violations	Rounds report implementation improved resident efficiency, increased time for direct patient care and education, and reduced duty-hour violations; no specific EHR time metrics in the abstract
Shenson et al., 2016 [39]	Surgical specialties using the patient portal	Surgeons	Retrospective portal visit analysis	Workload proxy	Secure messaging volume; clinic visits; proportion of outpatient interaction via secure messaging	Over 3 years, surgeons delivered 648,200 clinic visits and received 83,912 messages; monthly message volume grew >200%; proportion of outpatient interactions via messaging increased from 5.4% (2008) to 15.3% (2010)
Sun et al., 2018 [40]	Night float residents (multiple specialties)	Residents	Review and categorization of pages	Alarm fatigue, workload	Night-time pages to residents; urgency classification; content (updates, orders, labs)	Of 1820 pages, 62.1% were urgent and 27.7% non-urgent; non-urgent pages commonly for status updates, low-priority orders, and non-critical lab values
Cronin et al., 2015 [41]	Multiple specialties	Mixed providers, including surgeons	Retrospective portal visit analysis	Workload proxy	Patient-initiated secure messages and clinic visits; proportion of outpatient interactions via messaging	2,422,114 clinic visits and 948,428 messages over 3 years; medicine sent 78.3% of messages, surgery 8.9%; proportion of outpatient interactions via messaging increased from 12.9% (2008) to 39.8% (2010)
Congelosi et al., 2023 [42]	Surgical providers and patient portal	Surgeons	Survey before/after policy change	Burnout perceptions	Perceived impact of patient portal and 21st Century Cures Act on workload, messaging, and care	Describes changes in surgical providers’ perceptions of patient portal use and workload pre‑ vs. post‑policy; specific quantitative EHR–burnout effect estimates not provided in abstract
Crowson et al., 2016 [43]	Otolaryngology head and neck surgery inpatient providers	Residents	Observational study of tablet use	Efficiency/Workflow	Daily mobile tablet use to access EHR (notes, results, imaging) and support rounding	Demonstrated that tablet use was feasible and perceived as useful; reported improvements in perceived efficiency and access to information; specific EHR time metrics not given in the abstract
Freiburg et al., 2011 [44]	Surgical residency programs	Residents and surgeons/attendings	Survey of work hour restriction strategies	Education and workload outcomes	Program-level strategies (e.g., night-float, shift changes, task redistribution) affecting documentation and time demands	Describes strategies and programs used to accommodate duty-hour restrictions; provides context for time and documentation pressures; no EHR-specific quantitative metrics reported

## References

[1] Wolfe L, Chisolm MS, Bohsali F (2018). Clinically excellent use of the electronic health record: review. JMIR Hum Factors.

[2] Menachemi N, Collum TH (2011). Benefits and drawbacks of electronic health record systems. Risk Manag Healthc Policy.

[3] Amarasingham R, Plantinga L, Diener-West M, Gaskin DJ, Powe NR (2009). Clinical information technologies and inpatient outcomes: a multiple hospital study. Arch Intern Med.

[4] Bates DW, Leape LL, Cullen DJ (1998). Effect of computerized physician order entry and a team intervention on prevention of serious medication errors. JAMA.

[5] Tierney WM, Miller ME, McDonald CJ (1990). The effect on test ordering of informing physicians of the charges for outpatient diagnostic tests. N Engl J Med.

[6] Kucher N, Koo S, Quiroz R, Cooper JM, Paterno MD, Soukonnikov B, Goldhaber SZ (2005). Electronic alerts to prevent venous thromboembolism among hospitalized patients. N Engl J Med.

[7] Ledwich LJ, Harrington TM, Ayoub WT, Sartorius JA, Newman ED (2009). Improved influenza and pneumococcal vaccination in rheumatology patients taking immunosuppressants using an electronic health record best practice alert. Arthritis Rheum.

[8] Cotobal Rodeles S, Martín Sánchez FJ, Martínez-Sellés M (2025). Physician and medical student burnout, a narrative literature review: challenges, strategies, and a call to action. J Clin Med.

[9] Tajirian T, Stergiopoulos V, Strudwick G (2020). The influence of electronic health record use on physician burnout: cross-sectional survey. J Med Internet Res.

[10] Tawfik DS, Profit J, Morgenthaler TI (2018). Physician burnout, well-being, and work unit safety grades in relationship to reported medical errors. Mayo Clin Proc.

[11] Maslach C, Leiter MP (2016). Understanding the burnout experience: recent research and its implications for psychiatry. World Psychiatry.

[12] Rothenberger DA (2017). Physician burnout and well-being: a systematic review and framework for action. Dis Colon Rectum.

[13] Martinez KA, Sullivan AB, Linfield DT, Shaker V, Yu PC, Rothberg MB (2022). Change in physician burnout between 2013 and 2020 in a major health system. South Med J.

[14] Shanafelt TD, Hasan O, Dyrbye LN, Sinsky C, Satele D, Sloan J, West CP (2015). Changes in burnout and satisfaction with work-life balance in physicians and the general US working population between 2011 and 2014. Mayo Clin Proc.

[15] Dimou FM, Eckelbarger D, Riall TS (2016). Surgeon burnout: a systematic review. J Am Coll Surg.

[16] Dyrbye LN, Shanafelt TD, Balch CM, Satele D, Sloan J, Freischlag J (2011). Relationship between work-home conflicts and burnout among American surgeons: a comparison by sex. Arch Surg.

[17] Shanafelt TD, Balch CM, Dyrbye L (2011). Special report: suicidal ideation among American surgeons. Arch Surg.

[18] Balch CM, Freischlag JA, Shanafelt TD (2009). Stress and burnout among surgeons: understanding and managing the syndrome and avoiding the adverse consequences. Arch Surg.

[19] Li C, Parpia C, Sriharan A, Keefe DT (2022). Electronic medical record-related burnout in healthcare providers: a scoping review of outcomes and interventions. BMJ Open.

[20] Malay DS (2020). The burgeoning medical record. J Foot Ankle Surg.

[21] Cox ML, Risoli T Jr, Peskoe SB, Turner DA, Migaly J (2021). Quantified electronic health record (EHR) use by academic surgeons. Surgery.

[22] Ebbers T, Kool RB, Smeele LE, Takes RP, van den Broek GB, Dirven R (2022). Quantifying the electronic health record burden in head and neck cancer care. Appl Clin Inform.

[23] Dymek C, Kim B, Melton GB, Payne TH, Singh H, Hsiao CJ (2021). Building the evidence-base to reduce electronic health record-related clinician burden. J Am Med Inform Assoc.

[24] Yan Q, Jiang Z, Harbin Z, Tolbert PH, Davies MG (2021). Exploring the relationship between electronic health records and provider burnout: a systematic review. J Am Med Inform Assoc.

[25] Lilly CM, Cucchi E, Marshall N, Katz A (2019). Battling intensivist burnout: a role for workload management. Chest.

[26] Kesler K, Wynn M, Pugely AJ (2022). Time and clerical burden posed by the current electronic health record for orthopaedic surgeons. J Am Acad Orthop Surg.

[27] Somerson JS, Patton A, Ahmed AA, Ramey S, Holliday EB (2020). Burnout among United States orthopaedic surgery residents. J Surg Educ.

[28] Victores AJ, Coggins K, Takashima M (2015). Electronic health records and resident workflow: a time-motion study of otolaryngology residents. Laryngoscope.

[29] Coleman DM, Money SR, Meltzer AJ (2021). Vascular surgeon wellness and burnout: a report from the Society for Vascular Surgery Wellness Task Force. J Vasc Surg.

[30] McPeek-Hinz E, Boazak M, Sexton JB (2021). Clinician burnout associated with sex, clinician type, work culture, and use of electronic health records. JAMA Netw Open.

[31] Lin HL, Wu DC, Cheng SM, Chen CJ, Wang MC, Cheng CA (2020). Association between electronic medical records and healthcare quality. Medicine (Baltimore).

[32] Bahr TJ, Ginsburg S, Wright JG, Shachak A (2023). Technostress as source of physician burnout: an exploration of the associations between technology usage and physician burnout. Int J Med Inform.

[33] Ho VT, Sgroi MD, Chandra V, Asch SM, Chen JH, Lee JT (2023). Utilizing remote access for electronic medical records reduces overall electronic medical record time for vascular surgery residents. J Vasc Surg.

[34] Drudi LM, Mitchell EL, Chandra V (2022). A gender-based analysis of predictors and sequelae of burnout among practicing American vascular surgeons. J Vasc Surg.

[35] Carayon P, Wetterneck TB, Alyousef B (2015). Impact of electronic health record technology on the work and workflow of physicians in the intensive care unit. Int J Med Inform.

[36] Aziz F, Talhelm L, Keefer J, Krawiec C (2019). Vascular surgery residents spend one fifth of their time on electronic health records after duty hours. J Vasc Surg.

[37] Wormer BA, Colavita PD, Yokeley WT (2015). Impact of implementing an electronic health record on surgical resident work flow, duty hours, and operative experience. Am Surg.

[38] Ham PB 3rd, Anderton T, Gallaher R (2016). Development of electronic medical record-based "rounds report" results in improved resident efficiency, more time for direct patient care and education, and less resident duty hour violations. Am Surg.

[39] Shenson JA, Cronin RM, Davis SE, Chen Q, Jackson GP (2016). Rapid growth in surgeons' use of secure messaging in a patient portal. Surg Endosc.

[40] Sun AJ, Wang L, Go M, Eggers Z, Deng R, Maggio P, Shieh L (2018). Night-time communication at Stanford University Hospital: perceptions, reality and solutions. BMJ Qual Saf.

[41] Cronin RM, Davis SE, Shenson JA, Chen Q, Rosenbloom ST, Jackson GP (2015). Growth of secure messaging through a patient portal as a form of outpatient interaction across clinical specialties. Appl Clin Inform.

[42] Congelosi PD, Eid MA, Sorensen MJ (2023). Surgical providers' perceptions of the patient portal: before and after the 21st century Cures Act. J Surg Res.

[43] Crowson MG, Kahmke R, Ryan M, Scher R (2016). Utility of daily mobile tablet use for residents on an otolaryngology head & neck surgery inpatient service. J Med Syst.

[44] Freiburg C, James T, Ashikaga T, Moalem J, Cherr G (2011). Strategies to accommodate resident work-hour restrictions: impact on surgical education. J Surg Educ.

[45] Tapuria A, Porat T, Kalra D, Dsouza G, Xiaohui S, Curcin V (2021). Impact of patient access to their electronic health record: systematic review. Inform Health Soc Care.

[46] O'Malley AS, Grossman JM, Cohen GR, Kemper NM, Pham HH (2010). Are electronic medical records helpful for care coordination? Experiences of physician practices. J Gen Intern Med.

[47] Florig ST, Corby S, Rosson NT, Devara T, Weiskopf NG, Gold JA, Mohan V (2022). Chart completion time of attending physicians while using medical scribes. AMIA Annu Symp Proc.

[48] Kroth PJ, Morioka-Douglas N, Veres S (2019). Association of electronic health record design and use factors with clinician stress and burnout. JAMA Netw Open.

[49] Sinsky C, Colligan L, Li L (2016). Allocation of physician time in ambulatory practice: a time and motion study in 4 specialties. Ann Intern Med.

[50] Marckini DN, Samuel BP, Parker JL, Cook SC (2019). Electronic health record associated stress: a survey study of adult congenital heart disease specialists. Congenit Heart Dis.

[51] Khairat S, Coleman C, Ottmar P, Jayachander DI, Bice T, Carson SS (2020). Association of electronic health record use with physician fatigue and efficiency. JAMA Netw Open.

[52] Bohrer T, Koller M, Schlitt HJ, Bauer H (2011). Workload and quality of life of surgeons. Results and implications of a large-scale survey by the German Society of Surgery. Langenbecks Arch Surg.

[53] Gardner RL, Cooper E, Haskell J, Harris DA, Poplau S, Kroth PJ, Linzer M (2019). Physician stress and burnout: the impact of health information technology. J Am Med Inform Assoc.

[54] Campbell EM, Sittig DF, Ash JS, Guappone KP, Dykstra RH (2006). Types of unintended consequences related to computerized provider order entry. J Am Med Inform Assoc.

[55] Leigh JP, Tancredi D, Jerant A, Kravitz RL (2011). Annual work hours across physician specialties. Arch Intern Med.

[56] Kent GG, Johnson AG (1995). Conflicting demands in surgical practice. Ann R Coll Surg Engl.

[57] Davies LW, Dibbs RP, Appel R, Ferry AM, Buchanan EP (2021). The inefficiency of electronic medical record use by surgical healthcare professionals. JAAPA.

[58] Miake-Lye IM, Cogan AM, Mak S (2023). Transitioning from one electronic health record to another: a systematic review. J Gen Intern Med.

[59] Larsen E, Hoffman D, Rivera C, Kleiner BM, Wernz C, Ratwani RM (2019). Continuing patient care during electronic health record downtime. Appl Clin Inform.

[60] Kutney-Lee A, Brooks Carthon M, Sloane DM, Bowles KH, McHugh MD, Aiken LH (2021). Electronic health record usability: associations with nurse and patient outcomes in hospitals. Med Care.

[61] Dutta B, Hwang HG (2020). The adoption of electronic medical record by physicians: a PRISMA-compliant systematic review. Medicine (Baltimore).

[62] Uslu A, Stausberg J (2021). Value of the electronic medical record for hospital care: update from the literature. J Med Internet Res.

[63] Longhurst CA, Davis T, Maneker A (2019). Local investment in training drives electronic health record user satisfaction. Appl Clin Inform.

[64] Etheridge JC, Goldstone RN, Harrington B, Calcaterra MJ, Tomczyk EG, Parangi S, Haas S (2023). Implementation of a new surgeon onboarding program in an academic-affiliated community hospital. Ann Surg.

[65] Lourie EM, Utidjian LH, Ricci MF, Webster L, Young C, Grenfell SM (2021). Reducing electronic health record-related burnout in providers through a personalized efficiency improvement program. J Am Med Inform Assoc.

[66] Goss FR, Blackley SV, Ortega CA (2019). A clinician survey of using speech recognition for clinical documentation in the electronic health record. Int J Med Inform.

[67] Payne TH, Alonso WD, Markiel JA, Lybarger K, White AA (2018). Using voice to create hospital progress notes: description of a mobile application and supporting system integrated with a commercial electronic health record. J Biomed Inform.

[68] Bajwa J, Munir U, Nori A, Williams B (2021). Artificial intelligence in healthcare: transforming the practice of medicine. Future Healthc J.

[69] Bates DW, Landman AB (2018). Use of medical scribes to reduce documentation burden: are they where we need to go with clinical documentation?. JAMA Intern Med.

[70] Yan C, Rose S, Rothberg MB, Mercer MB, Goodman K, Misra-Hebert AD (2016). Physician, scribe, and patient perspectives on clinical scribes in primary care. J Gen Intern Med.

[71] Gottlieb M, Palter J, Westrick J, Peksa GD (2021). Effect of medical scribes on throughput, revenue, and patient and provider satisfaction: a systematic review and meta-analysis. Ann Emerg Med.

[72] Gao RW, Dugala A, Maxwell J, Falconer P, Birkeland AC, Divi V, Rosenthal EL (2020). Effect of medical scribes on outpatient oncology visits at a multidisciplinary cancer center. JCO Oncol Pract.

[73] Cho J, Sanchez K, Ganor O, Afshar S, Ruditsky A, Bierman A, Taghinia AH (2019). Utilizing a physician scribe in a pediatric plastic surgical practice: a time-driven activity-based costing study. Plast Reconstr Surg Glob Open.

[74] Gyimah MB, Shah HP, Lee YH (2022). Maximizing the effectiveness of scribes in surgical practices. Am J Surg.

